# Rethinking (again) Hardy-Weinberg and genetic drift in undergraduate biology

**DOI:** 10.3389/fgene.2023.1199739

**Published:** 2023-06-08

**Authors:** Michael W. Klymkowsky

**Affiliations:** Molecular, Cellular, and Developmental Biology University of Colorado Boulder, Boulder, CO, United States

**Keywords:** genetic drift, stochasticity, Hardy-Weinberg equilibria, course design, biology education

## Abstract

Designing effective curricula is challenging. Content decisions can impact both learning outcomes and student engagement. As an example consider the place of Hardy-Weinberg equilibria (HWE) and genetic drift calculations in introductory biology courses, as discussed by Masel (2012). Given that population genetics, “a fairly arcane speciality”, can be difficult to grasp, there is little justification for introducing introductory students to HWE calculations. It is more useful to introduce them to the behavior of alleles in terms of basic features of biological systems, and that in the absence of selection recessive alleles are no “weaker” or preferentially lost from a population than are dominant alleles. On the other hand, stochastic behaviors, such as genetic drift, are ubiquitous in biological systems and often play functionally significant roles; they can be introduced to introductory students in mechanistic and probabilistic terms. Specifically, genetic drift emerges from the stochastic processes involved in meiotic chromosome segregation and recombination. A focus on stochastic processes may help counteract naive bio-deterministic thinking and can reinforce, for students, the value of thinking quantitatively about biological processes.

## Introduction

One might suppose that college level biology degree programs are designed via an intelligent (backward) design process (Wiggins and McTighe, 1998), shaped by a program’s desired learning outcomes. Learning outcomes are presumed to prepare students for the demands of post-graduate life and careers. Such a design process can be expected to anticipate and address necessary challenges and to avoid unnecessary obstacles. The actual curricular design process is, however, rarely so rational and goal oriented and often fails to re-consider content covered in the light of new experimental insights. Calls for “active learning” pedagogical strategies (Wieman, 2017; 2014) are often imposed on top of unaltered course content and fail to acknowledge the challenges associated with more interactive and discussion-oriented approaches. More broadly, a program’s learning goals are often only broadly sketched out or assigned to be “covered” in specific courses.

An obvious question then is whether the courses addressing these topics lead to students who understand their implications and application? In a number of cases related to chemistry (Lewis Structures, H-bonds, Acid-Base, London-dispersion forces) direct observations indicate that conventional curricula often fail to produce the kind of student understanding presumed to have occurred (see as examples Becker et al., 2016; Cooper et al., 2010; Cooper et al., 2016; Noyes and Cooper, 2019). In a classic example, a conventional chemistry curriculum (taught in an “active learning” style) can leave students confused as to the differences between H-bonds and covalent bonds involving hydrogen (Williams et al., 2015). Such confusions are likely to impact understanding of more advanced topics that involve chemical bonds and molecular interactions. Recent analyses indicate that conventional chemistry courses often focus more on mathematical calculations than on chemistry’s conceptual foundations (Stowe and Cooper, 2017; Stowe and Cooper, 2019; Stowe et al., 2021; Ralph et al., 2022). In the context of an introductory course, such a disconnect can suggest that the purpose of the course is to sort rather than educate students (see Mervis, 2011; Hatfield et al., 2022). Such a perception may impact (non-privileged) students who may lack the “social capital” needed to place the course in context (Israel et al., 2001; Rogošić and Baranović, 2016).

In the biological sciences there is a tacit presumption that students need courses in (macroscopic) physics, chemistry, and mathematics to be able to understand the workings of biological systems (see the National Research Council’s Bio2010 report (NRC, 2003) and Vision and Change (AAAS, 2011; Brownell et al., 2014)). Not withstanding calls for integrating the mathematics needed to model and analyze complex systems (Bialek and Botstein, 2004; Wingreen and Botstein, 2006; Labov et al., 2010) it remains unclear exactly which mathematical/analytic skills will be useful going forward and how they might be best introduced to students. For example, what background training is required to understand the behavior of a gene network or make sense of the results of an analysis of a multi-dimensional data set such as produced by single cell RNA sequencing or genome-wide association studies? In the context of such complex data sets are web-based tutorials on “Principle Component Analysis for Dummies” (LINK) and “Making sense of principal component analysis, eigenvectors & eigenvalues” (LINK) sufficient, or is a more complete rethinking of course and curricular content necessary and for whom? As outlined previously, at minimum there needs to be an appreciation of how varying the inputs into a model can lead to dramatic differences in system behaviors (Klymkowsky, 2021).

## Institutional factors influencing introductory biology course design

While there is typically a single physics or chemistry department, there can be multiple departments (or divisions) related to biological systems. The result can be a lack of consensus as to what exactly the introductory course sequence should focus on. An obvious factor that influences curricular design are the post-graduation employment and educational paths that students aim to pursue. As an example course requirements for biology degree programs are often influenced by medical school entrance requirements and exams. One outcome is the “survey” course, a course that touches, sometimes quite superficially, on a wide range of complex topics. The value of these courses has been discussed and recommendations made (see Becker, 2005; Klymkowsky et al., 2016b; Ledbetter and Campbell, 2005 as examples) but clearly the exact focus of such courses depend upon the specific goals of the degree program involved. In part the situation arises from the fact that biology lacks the universally recognized “laws” that provide a foundation for physics and chemistry (Mayr, 1985). Biological systems (cells, organisms, populations, species) are emergent objects shaped by unpredictable historical “accidents” and events; these include mutations, environmental changes and catastrophes, migrations, pathogens and predators, and events that lead to the reproductive isolation of populations from one another, the basis for speciation–the subject of Darwin’s (1859) “Origin of Species”.

While the diversity of life is clearly a critical aspect of biological studies, there is a fundamental unity that underlies all biological considerations. All existing evidence indicates that all known organisms share a “last universal common ancestor” (LUCA). The major molecular processes and cellular characteristics of biological systems are conserved (albeit with modifications): basic cell structure, the processes involved in capturing and encoding information in DNA and then “expressing” that information through regulated RNA and RNA-directed polypeptide synthesis, together with core metabolic processes through which energy is captured and used to drive the thermodynamically unfavorable reactions involved in growth, adaptation, and the maintenance of the non-equilibrium living state. This point was made by Gerhart and Kirschner (1997) and reinforced by more recent observations, on the small number of basic processes that underlie the morphological and behavioral diversity of living systems. In this light, focussing an introductory biology course sequence on these common processes and principles would seem to make sense.

## Do Hardy-Weinberg equilibrium (HWE) calculations have a place in an introductory biology (or genetics) course?

Masel (2012) appears to hold that HWE calculations are an essential component of introductory biology (and genetics) courses. A perusal of various recent introductory biology/genetics textbooks (e.g., codon learning - link) include HWE calculations. There are a number of published papers on how best to implement such calculations within a course.^1^ As described by Masel, HWE calculations rely heavily on mathematical concepts that may be poorly understood by students, such as the concept of the null hypothesis and the use of statistical tests to evaluate whether observations support the null hypothesis. It therefore is worth considering 1) the assumptions that HWE calculations are based on, 2) what general conclusions arise from these calculations, at least at the introductory level, and 3) when, going forward, will students be called upon to carry out such calculations? The HWE framework rests on a number of unrealistic assumptions leading to an equilibrium state; these assumptions are 1) the absence of new mutations in the population, 2) the absence of selection of all types, 3) random (non-selective) mating, 4) the absence of migration between populations, and v) an infinitely large population in which genetic drift does not occur.

“There is little point in teaching a biology concept before the students have sufficient math to understand it. Masel (2012).

So, outside of the context of more advanced population genetic studies, what can an introductory student conclude from HWE calculations? The key take-away is that, in the absence of selection, recessive mutations/alleles are no more likely to be lost from a population than are dominant mutations/alleles. Recessive alleles are not “weaker” than dominant alleles. As far as I can discern, there is little or no observational evidence that this fact is widely appreciated by students. This is relevant in the light of concept inventory-based observations that students are confused as to the behaviors of recessive and dominant alleles (Klymkowsky et al., 2010; Champagne-Queloz et al., 2017). An alternative to HWE calculations might be to follow a more historical approach, based on the relationships between phenotype and selection (e.g., Sanjak et al., 2018) and the work of H.J. Muller (2016; 1932) in which alleles (and by implication mutations) are defined at the functional and phenotypic level based on differences from “wild type” alleles (see Henson et al., 2012). At the same time, this involves recognizing that a particular allele can be recessive with regard to one phenotypic trait and dominant for another. Alleles associated with sickle cell anemia display this type of recessive and dominant behavior.

Students are often assessed on their ability to carry out HWE calculations rather than on their understanding of how allelic variations can influence phenotype, something that is often complex and that transcends the typical Mendelian framing of genetics (see Klymkowsky, 2022 review of Haskel-Ittah and Yarden Putting Genetics in Context). While the relevance of HWE calculations to understanding the phenotypic effects of genetic changes is not immediately clear, a focus on calculation for the sake of calculation can favor those with previous mathematical training, but may have no benefit in terms of improved conceptual understanding (Stowe and Cooper, 2019; Ralph et al., 2022).

## The origins of genetic drift and the stochastic nature of biological systems

The molecular and cellular organization of biological systems leads to stochastic behaviors that often go unconsidered in “conventional” courses. For many stochasticity is a new term, often confused with randomness, which is different, and noisiness, which is an aspect of stochastic behaviors (for reviews see Allen, 2010; Bressloff, 2014; Honegger and de Bivort, 2018). A truly random process would be both unpredictable and display no overall pattern. From a strictly naturalistic perspective, random events do not occur. A stochastic event, such as the decay of a radioactive atom, while not predictable at the level of the individual atom (the basis of Schrȍdinger’s cat thought experiment), is lawful. Given a large enough population of atoms, we can accurately predict the time at which 50% will have decayed. Some will have decayed much earlier and some very much later. In biological systems, stochasticity arises in large part from thermal motion, which impacts the formation and dissociation of molecular interactions and chemical reactions. Molecular collisions can deliver and take away kinetic energy. The exact momentum of each molecule cannot, however, be accurately and completely characterized without perturbing the system.

The small size of most cells, and the fact that there are small numbers of key molecules within them (for example, DNA) means that stochastic processes play an important role in biological behaviors and their regulation; stochastic changes in gene expression can have profound effects on the future of the cell (and the organism). The ubiquity of stochastic effects and responses to these effects has been revealed most recently through single cell gene expression and RNA sequencing methods (e.g., Elowitz et al., 2002; Chess, 2016; Briggs et al., 2018) and by following the behavior of cells whose behavior, in terms of gene expression, has diverged from their neighbors (community effects) (see as examples Di Gregorio et al., 2016; Akieda et al., 2019; Morata, 2021).

Some confusions appear to exist within the biology education community about the nature of stochastic processes. For example, Price et al. (2014) describe a “misconception” uncovered in the course of the construction of their “Genetic Drift Concept Inventory”. They note that “students conflate randomness with unpredictability”; they quote a student who stated, “because genetic drift is random, you will not know when the genes will drift until it is done, so you can’t predict it”. But this is no misconception, it is exactly correct - the molecular events that produce the genotype of a specific gamete and that lead to genetic drift in small populations are not predictable. These changes become increasingly predictable as population size increases. This same rule applies to the chemotactic tumbling of bacteria (Spudich and Koshland, 1976), the noisy opening of ion channels (Neher and Sakmann, 1976), patterns of cell differentiation, interactions, and activities in the immune (Abadie et al., 2019) and nervous (Rolls and Deco, 2010; Rolls, 2016) systems, and gene expression in general (Elowitz et al., 2002; Vilar et al., 2003; Choi et al., 2008; Deng et al., 2014; Gendrel et al., 2014; Reinius and Sandberg, 2015; Chess, 2016) and a range of other phenomena (Honegger and de Bivort, 2018; You and Leu, 2020; Klymkowsky, 2023). While the behavior of (a large enough) population is predictable, the behavior of any single actor is not. It is in this context that various assessment instruments (Garvin-Doxas and Klymkowsky, 2008; Klymkowsky et al., 2016a; Champagne-Queloz et al., 2017; Tobler et al., 2023) can be useful in identifying when students have learned to apply ideas appropriately.

Genetic drift, that is, non-adaptive changes in allele frequencies, is called out by Masel (2012) as a key concept. In Masel’s view, genetic drift arises from “accidents of sampling”, a framing intrinsic to population biology and evolution studies. In common parlance, sampling is something done to a pre-existing population, as occurs in the context of founder and bottleneck effects. In these situations small numbers of individuals are taken from a larger population and then evolve independently. Sampling does not address the origins of the allelic variation found within the population, which are due to mutation and recombination, the raw material on which genetic drift acts. No population is sampled to produce the variation in gametes and embryos.

During sexual reproduction each new organism is the product of at least three distinct stochastic events.^2^ During the first phase of meiosis, homologous maternal and paternal chromosomes align and come to lie at the metaphase plate of the meiosis I spindle. With anaphase I, each daughter cell receives either a maternal or a paternal copy of each chromosome, but which copy they receive is unpredictable, and based on the stochastic orientation of maternal and paternal chromosome kinetochores (Hauf and Watanabe, 2004). The situation is further complicated by the occurrence of crossing over (recombination) events that “shuffle” the maternal and paternal regions of homologous chromosomes. The result is a series of independent “coin-flip decisions” that determine the chromosomal “identity” of the resulting cells. In humans, with 23 distinct chromosomes, there are 2^23^ possible outcomes without considering the effects of recombination, which greatly increases the number of possible outcomes. With the second meiotic division four haploid cells are generated with two (out of >2^23^ possible) distinct maternal/paternal chromosome compositions. A similar process occurs in the paternal germ line. Which female gamete fuses with which male gamete to form a diploid embryo is again stochastic. The result is that each new individual is unique, i.e., not predictable based on the genotypes of its parents—assuming that we are not dealing with the unnatural situation of fully inbred parents. By considering the origins of the genetic variation among gametes and embryos, students can be introduced to the impact of stochastic processes in biological systems.

## Conclusions and recommendations

The basic question is whether introducing students to isolated aspects of population genetics and the mathematical analysis of complex allelic behaviors arising from interactions between genes, environments, and other organisms serves any purpose for the early (introductory) stage student. Instead a focus on the core ideas involved, the behaviors of alleles and the ubiquity of stochastic processes in biological systems seems more appropriate and useful. Understanding why stochastic “noise” occurs, and how it is utilized and controlled provides a context within which to make sense of the, often unpredictable variations in the behavior of organisms, including people.

## Data Availability

The original contributions presented in the study are included in the article/Supplementary Material, further inquiries can be directed to the corresponding author.

## Footnotes

1 A partial list of such papers can be found in reviewer #1’s comments on the first version of this manuscript.

2 For simplicity’s sake, I will consider only common variants of these processes, even though there are often exceptions and species specific variants.
